# Surgical management of a rare giant ovarian serous cystadenoma with distinctive clinical manifestations

**DOI:** 10.1093/jscr/rjad194

**Published:** 2023-06-05

**Authors:** Nima Shokrollahi, Mohammad Nouri, Alireza Movahedi, Fereshteh Kamani

**Affiliations:** Student Research Committee, School of Medicine, Shahid Beheshti University of Medical Sciences, Tehran 1983969411, Iran; Student Research Committee, School of Medicine, Shahid Beheshti University of Medical Sciences, Tehran 1983969411, Iran; Department of General Surgery, School of Medicine Ayatollah Taleghani Hospital, Shahid Beheshti University of Medical Sciences, Tehran 1983969411, Iran; Department of General Surgery, School of Medicine Ayatollah Taleghani Hospital, Shahid Beheshti University of Medical Sciences, Tehran 1983969411, Iran

## Abstract

Giant ovarian cysts are categorized with sizes >10 cm. After reaching huge diameters, these rare tumors cause clinical symptoms such as nausea, vomiting or abdominal pain. We present a 29-year-old woman with a giant unique cystadenoma represented with unusual clinical features like low back pain and progressive constipation. Specific imaging methods demonstrated an adnexal lesion in the form of an enormous ovarian cyst; afterward, an open laparotomy was recommended to access the abdominal cavity. The critical role of timely diagnosis and accurate workups for giant ovarian cysts in increasing patients’ life expectancy and quality of life is discussed.

## INTRODUCTION

Around 7% of women worldwide are diagnosed with an ovarian cyst in their lifetime [1]. The prevalence of these cysts is 6.6% in premenopausal women but increases up to 18% after their menopause [2, 3]. Ovarian cysts, either simple or complex, are usually found benign, although complex ovarian masses have more chance to be malignant [4]. These rare tumors can only be symptomatic after passing the early stages and reaching huge diameters. The clinical symptoms such as nausea, vomiting or non-specific abdominal pain are related to the pressure that a giant cyst can cause, which means larger cysts have more symptoms [5]. Therefore, they must be resected to eliminate the risk of malignancy and symptoms [6]. In this article, we present a case of uncommon serous cystadenoma in a premenopausal woman with rare clinical features, which was detected with imaging studies and removed surgically.

## CASE PRESENTATION

A 29-year-old woman was admitted to our hospital with the chief complaint of sudden onset of pain located mainly on the low back and right flank radiating to the hypogastric region during the previous week. She also has the same pain that lasted 2 days a month ago. The pattern of pain was on and off but was relieved with rest. The patient had nausea and vomiting at the onset of the pain; also, she mentioned constipation that improved over time with severe anorexia. The physical examination represented a pulse rate of 80 beats/min, a respiratory rate of 18 breaths/min and blood pressure of 110/70 mmHg. Laboratory results demonstrated hemoglobin of 9.5 g/dl, hematocrit of 30.2, carcinoembryonic antigen (CEA) of 9.0 (Table 1) and cancer antigen 15–3 of (CA 15–3) 71.

**Table 1:** Perioperative clinical profile of the patient with normal values

Name	Result	Normal range
CEA	**9.0**	$<5\mathrm{ng}/\mathrm{ml}$
CA 15–3	**71**	$<40\mathrm{U}/\mathrm{ml}$
CA 125	13.0	$<35\mathrm{U}/\mathrm{ml}$

The high values of tumor markers in the patient's antigen tests: carcinoembryonic antigen (CEA) and cancer antigen 15–3 (CA 15–3) are higher than the normal level. However, the cancer antigen 125 (CA–125) level is normal.

**Figure 1:**
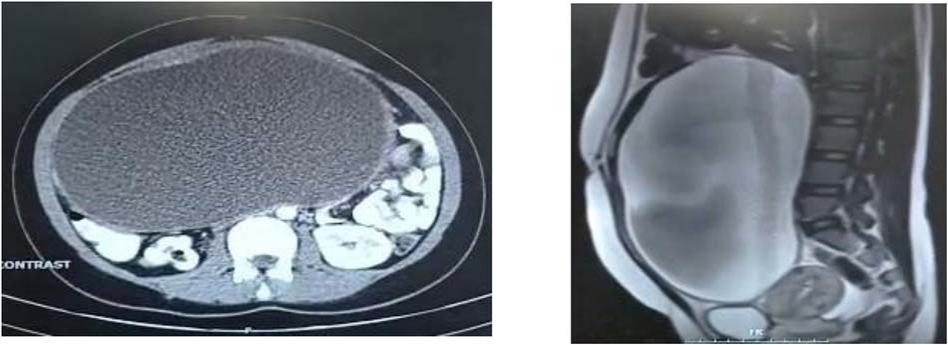
CT scan of the abdomen represents an extension of the mass in two specific views. (**a**) From the front of the lumbar vertebra to the abdominal wall in horizontal view. (**b**) Below the peritoneal cavity to the above the bladder in sagittal view.

The patient had an ultrasound which demonstrated a cystic lesion with dimensions of 220 × 120 mm. Computed tomography (CT) scan and magnetic resonance imaging of the abdomen and pelvis were requested for further examination, which revealed an adnexal lesion in the form of a giant ovarian cyst (Fig. 1).

**Figure 2:**
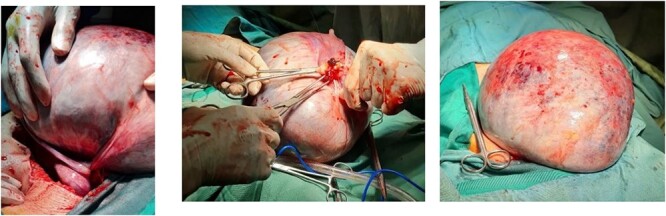
The appearance of massive abdominal mass after tumor excision. (**a**) Ovarian torsion has occurred secondary to the cyst, along with dark lines indicating a hemorrhagic infarction. (**b**) Ovarian cystic mass cut from the origin. (**c**). Dimensions of ovarian cyst.

Based on the possible diagnosis of an ovarian cyst due to the large size of the cyst, an open laparotomy was recommended to access the abdominal cavity. If malignancy is reported, total abdominal hysterectomy with bilateral salpingo-oophorectomy is needed to prevent the tumor from progressing. During surgical exploration, ovarian torsion was found due to the large volume of the cyst (Fig. 2a). After removing the mass, a frozen sample was sent to the pathology department, and it was revealed that the tumor was benign. This mass consists of a cystically dilated ovarian tissue measuring 28 × 21 × 12 cm^3^ with an attached fallopian tube measuring 10 × 1 cm^2^ (Fig. 2b). The tumor was identified as benign serous cystadenoma based on pathological findings. In the end, the patient was discharged from the hospital in proper condition and was scheduled to follow-up.

## DISCUSSION

Ovarian cysts are very prevalent findings in women during their lifetime, which often decrease in size or even disappear on their own over time. On the other hand, giant ovarian cysts are a very uncommon condition that has been reported in various sizes up to 148.6 kg by Spohn in 1922 [7]. Serous cystadenomas are benign ovarian epithelial tumors responsible for 20–50% of ovarian neoplasms and are most commonly diagnosed from 40 to 60 years [8, 9]. In our case, ovarian torsion was the cause of the patient’s low back pain and the main reason for referral. Imaging is a helpful method that leads us to proper diagnosis and is recommended in the initial assessments. In addition to initial clinical evaluation and imaging findings, tumor markers are beneficial tools to distinguish benign from malignant tumors.

To summarize everything that has been expressed so far, determining a giant ovarian cyst as a differential diagnosis in female patients with only weight loss and constipation or nonspecific symptoms such as low back pain is crucial for any practitioner.

## Data Availability

The data underlying this article are available in the article.
